# CXCL12/CXCR4 axis plays pivotal roles in the organ-specific metastasis of pancreatic adenocarcinoma: A clinical study

**DOI:** 10.3892/etm.2012.631

**Published:** 2012-07-04

**Authors:** WEIXIA ZHONG, WEIWEI CHEN, DEXIAN ZHANG, JUJIE SUN, YUHUI LI, JIANBO ZHANG, YONGSHENG GAO, WUYUAN ZHOU, SHENG LI

**Affiliations:** 1 Shandong Tumor Hospital, Jinan 250117;; 2 Jinan Central Hospital, Jinan 250001, P.R. China

**Keywords:** chemokines, chemokine receptors, organ-specific, pancreatic neoplasms

## Abstract

Pancreatic cancer is one of the most lethal types of cancer, and curative resection is only applicable to potentially limited cases due to early metastasis and local invasion. This study reports the influence of CXCL12 and its receptor CXCR4 on the progression of pancreatic cancer and highlights the correlation between the CXCL12/CXCR4 axis and the organ-specific metastasis of pancreatic adenocarcinoma (PAC). A total of 34 patients with pancreatic cancer participated in the current study. The expression of CXCL12 and CXCR4 in cancerous tissues, paracancerous tissues, normal pancreas and lymph nodes surrounding the pancreas were investigated using immunohistochemistry and RT-PCR; furthermore, their relationship with clinicopathological factors was explored (PV9000 method). The positive rate of CXCL12 protein was 13.3% (4/30), the positive rate of CXCR4 protein was 80% (24/30) in tumor tissues. Additionally, a significant correlation between the expression pattern of the CXCL12/CXCR4 axis with lymph node metastasis was identified (P<0.05), excluding gender, age, tumor node metastasis (TNM) stage and differentiation (all P>0.05). Also, the positive rate of CXCL12 protein was 50% (15/30), the positive rate of CXCR4 protein was 73.3% (22/30) in the lymphocytes in lymph nodes surrounding the pancreas. Furthermore, we found that CXCL12 and CXCR4 expression in paratumorous vessels and neural tissue were significantly strongly positive. The paratumorous vessels and neural tissue with positive CXCL12 and CXCR4 expression were invaded by CXCL12-positive pancreatic cancer cells. The chemotactic interaction between CXCR4 and its ligand CXCL12 may be a critical event during the progression of pancreatic cancer. The CXCL12/CXCR4 axis plays an important role in the progression and organ-specific metastasis of pancreatic adenocarcinoma.

## Introduction

As a relatively common gastroenterological malignant tumor, pancreatic adenocarcinoma (PAC) accounts for ∼8–10% of all gastroenterological malignancies and its incidence increases 15% every 10 years (1). Lymphatic metastasis is an important prognostic and treatment factor in PAC; post-mortem examination often reveals wide involvement of adjacent organs in PAC patients. Local lymphatic metastases in mesenteric lymph nodes, (lymph nodes next to the superior mesenteric artery), gastroduodenal artery, hilar and portal vein lymph nodes comprise the majority of lymphatic metastases. This may be due to the anatomic position and structure of the pancreas. Most of the pancreatic blood supply comes from the hepatic artery and mesenteric artery superior (2). Retroperitoneal and left supraclavicular lymph node metastases are also common, similar to other gastroenterological malignancies. Lymphatic spread is also found in up to 50% of so-called early cancer of the pancreas with the presence of metastases to adjacent or distant lymph nodes (3). Tumor cells can spread multi-directionally via lymph nodes, and pancreatic lymph reflux is part of the entire gastroenterological lymph reflux, having direct or indirect association with adjacent organs; this indicates the anatomic reason for the high metastatic property of PAC. The metastasis and invasion of PAC may occur via blood vessels, lymph node and direct spreading simultaneously.

CXCL12 is a member of the CXC chemokine subfamily, working together with its chemokine receptor CXCR4 (4). CXCL12 can induce the adherence of most circulating lymphocytes and pre-CD34 cells, and the secreted protein on the cell surface. It can interact with integrin, causing cells to migrate to a specific location (5). Endothelial cells express CXCR4, and CXCL12 confers a strong chemostatic effect on them (6). CXCR4 expression is unregulated in some breast cancer cells. CXCR4 antagonist can downregulate its expression in breast cancer cells, thus repressing metastasis in animal models (7,8). Kato *et al* (9) analyzed 79 cases of resected invasive ductal carcinoma samples, all cancerous tissue expressing CXCR4. Samples with high expression of CXCR4, particularly local high expression of CXCR4, often had extensive lymph node metastasis, indicating that CXCR4 may play pro-metastatic roles in the lymphatic metastasis of breast cancer. Administration of the monoclonal antibody to CXCR4 to SCID mice was found to effectively regress lung metastasis from MDA-MB-231 xenografts, indicating the key roles of chemokines and chemokine receptors in the organ-specific metastasis of breast cancer. Liang *et al* (10) found that CXCL12 caused a 2.2-fold increase in filamentous actin in breast cancer cells within 20 sec *in vitro*, resulting in the formation of pseudopods, induction of targeted migration and invasion of breast cancer cells, which was dose-dependent. The anti-CXCR4 antibody can block this effect. A better understanding of the molecular mechanisms of the relationship between lymphangiogenesis and lymphatic metastasis will provide the theoretical basis for the blockage of lymphatic metastasis, which has important clinical significance. To date, the clinical significance of the CXCL12/CXCR4 axis in pancreatic cancer has not yet been clearly elucidated. Thus, in the present study, we explored the molecular roles of the CXCL12/CXCR4 axis in the organ-specific metastasis of PAC.

## Materials and methods

### Patients and tissue samples

Tissue samples were obtained from 30 patients who underwent macroscopically curative resection for pancreatic cancer at Shandong Tumor Hospital (Jinan, China) between 2005 and 2007. Samples of the pancreatic tumor, paracancerous tissues, normal pancreas and lymph nodes surrounding the pancreas were immediately frozen in liquid nitrogen or formalin-fixed after surgery and then embedded in paraffin. Sections from each case were stained with hematoxylin and eosin (H&E) for histological examination according to the tumor node metastasis (TNM) classification system. Tissue was collected based on the protocol approved by the Ethics Committee of the Medical Faculty of Shandong Tumor Hospital (Jinan, China).

All patients had complete clinical and pathological data. The patients were comprised of 17 men and 13 women with a median age of 57.2 years (range, 35-78). No patients received preoperative chemotherapy or radiotherapy. Among the 30 patients, 17 well-differentiated and 13 poorly differentiated cases were identified. The study included 12 Union for International Cancer Control (UICC) stage I–II patients and 18 who were stage III–IV.

### Immunohistochemical staining

Tissue sections were analyzed using the standard protocol for streptavidin-peroxidase immuno histochemical staining. Sections (4-μm) were deparaffinized and rehydrated. After blocking of endogenous peroxidase with methanol containing 0.3% H_2_O_2_, the sections were autoclaved at 121°C for 10 min in a citrate buffer (10 mmol/l sodium citrate; pH 6.0) for antigen retrieval. After blocking with normal goat serum, the sections were incubated overnight with the following primary antibodies: CXCL12 (1:200), CXCR4 (1:200), VEGFR-3 (1:250) and CD34 (1:80) (DakoCytomation, Glostrup, Denmark). The sections were then reacted sequentially with biotin-conjugated anti-mouse immunoglobulin G antibodies (Vector Laboratories, Inc., Burlingame, CA) and Vectastain Elite ABC reagent (Vector Laboratories, Inc.). Diaminobenzidine was used as the chromogen, and the nuclei were counterstained with hematoxylin. One breast cancer sample was used as a positive control and phosphate-buffered solution (PBS) without the primary antibody was employed as the negative control. Each section was analyzed in terms of the staining intensity and the proportion of positive tumor cells by two pathologists in a double-blinded manner. The upper quartile was defined as the cutoff point.

Microvessels were detected by morphological observation and immunohistochemical labeling using the endothelial marker CD34 for microvascular density (MVD) evaluation. All independent CD34-positive vessels were counted regardless of the presence of an identifiable lumen. The following methods were employed to assess MVD. First, the area with the most intense vascularization was determined under magnification, ×10. The average MVD was then analyzed by randomly selecting 5 fields/tumor at magnification, ×400. Blood vessels with lumens containing >8 red cells or with muscular layers were excluded. For each case, the number of CD34-positive vessel structures in 5 high power fields was recorded, and the average value was considered as the MVD. The immunostaining results were assessed by two pathologists who were blind to the clinicopathological findings. Meanwhile, VEGFR-3 was used for micro-lymphatic vessel density (MLVD) evaluation. The process used to calculate MLVD was the same as that for the MVD assay discussed previously.

### RT-PCR analysis

Reverse transcript-PCR was performed according to the standard protocol. In brief, RNA extraction from frozen human specimens was performed using the acid guanidinium thiocyanate method. RNA was dissolved in diethylpyrocarbonate (DEPC)-treated water. The prepared RNA (1 μg) was mixed with reverse transcription reagents at a total volume of 20 μl and incubated for 30 min at 42°C to produce first-strand cDNA. A total of 1 μl cDNA was used for PCR amplification. The primer sequences were as follows: CXCR4, F, 5′-AGCTGTTGGTGAAAAGGTGGTCTATG-3′ and R, 5′-GCGCTTCTGGTGGCCCTTGGAGTGTG-3′; CXCL12, F, 5′-CCGCGCTCTGCCTCAGCGACGGGAAG-3′ and R, 5′-CTTGTTTAAAGCTTTCTCCAGGTACT-3′; β-actin, F, 5′-GTGGGGCGCCCCAGGCACCA-3′ and R, 5′-CTC CTTAATGTCACGCACGATTT-3′.

Following the manufacturer’s instructions, reverse transcription was performed at 42°C in the presence of 5 units AMV reverse transcriptase and 1 μg RNA for 60 min. AMV RT inactivation and RNA/cDNA/primer denaturation were performed at 95°C for 5 min to activate modified Taq polymerase followed by 40 cycles at 95°C for 10 min, 95°C for 10 sec and 56°C for 1 min and 1 cycle at 72°C for 35 sec. The PCR product was separated by 2% agarose gel electrophoresis. The gels were viewed using UV transillumination and photographed by a Kodak 120 gel imaging system.

### Statistical analysis

Data are shown as mean ± SE and analyzed by the SPSS software program (version 13.0 for Windows; SPSS, Inc., Chicago, IL). Comparison among different groups was performed by the Chi-square test, the Fisher’s exact, one-way ANOVA and Spearman’s rho tests. A value of P<0.05 was considered statistically significant.

## Results

### CXCL12 and CXCR4 expression in pancreatic cancer

**Figure 1:**
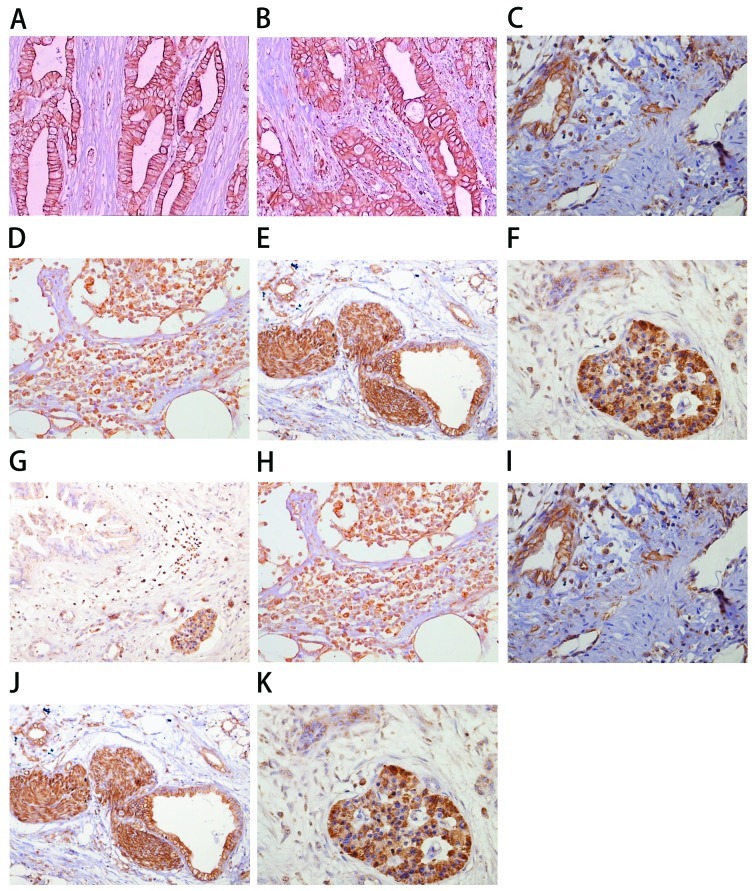
Immunohistochemical staining of CXCL12 in (A) normal pancreas, (B) cancerous tissue, (C) paracancerous tissue and (D) lymph node. Immunohistochemical staining of CXCR4 in (E) normal pancreas, (F) cancerous tissue, (G) paracancerous tissue, (H) lymph node, (I) blood vessels of cancerous tissue, (J) peripancreatic neural tissue and (K) an ‘island’ of cancerous tissue. Magnification, ×100.

The levels of CXCL12 and CXCR4 mRNA in the primary tumor, paracancerous tissues, normal pancreas and lymph nodes surrounding the pancreas were assessed using RT-PCR. Immunohistochemical staining was performed to further determine the levels and locations of CXCL12 and CXCR4 protein expression (Fig. 1). The rate of positive expression of CXCL12 was lower in the pancreatic tumor tissues compared with the positive rate in the other tissues (Table I). As shown in Table I, 80.0% of cancerous tissues (Fig. 1B), 70.0% of paracancerous tissues (Fig. 1C) and 73.3% of lymph node tissues (Fig. 1D) adjacent to the pancreas had positive expression of CXCR4. These results were significantly different from that of the normal pancreas tissue (Fig. 1A) (P<0.001). Positive expression of CXCR4 was also detected in the vascular endothelial cells of the pancreas (Fig. 1I), the peripancreatic lymph nodes (Fig. 1H), the peripancreatic neural tissue (Fig. 1J) and an ‘island’ of pancreatic cancer (Fig. 1K).

The level of CXCL12 mRNA was low in tumor tissues and moderate in the normal pancreas, while expression of CXCL12 was found in pancreatic cancer. A significant difference was found between the pancreatic cancer and normal pancreas (P<0.01) (Tables I and II). Moderate expression of CXCL12 was observed in the paracancerous tissues adjacent to the pancreas at the mRNA level, and this was significantly different from that of the pancreatic cancer group (P<0.05) (Table II). CXCL12 mRNA was detected at significantly high levels in paracancerous tissues, the normal pancreas and lymph nodes (Table II). The levels of CXCL12 protein exhibited the same trend as those of the mRNA (Fig. 1A–F). CXCR4 expression tended to be opposite that of CXCL12 at both the mRNA and protein levels (Tables I and II). Compared to the normal pancreas, the expression of CXCR4 in cancerous tissues, paracancerous tissues and lymph nodes was significantly higher (P<0.001) (Table II).

### Correlation of CXCL12 or CXCR4 expression with the clinicopathological factors of pancreatic cancer

To define the role of CXCL12 and CXCR4 in the progression of pancreatic cancer, we analyzed the association of expression profiles of CXCL12 and CXCR4 with tumor grade and stage. This analysis revealed that higher CXCR4 expression and cytoplasmic localization of CXCR4 were significantly associated with the lymph node status of the tumor (Table III). In addition, CXCR4 expression also correlated with stage in pancreatic cancer patients (P=0.05) (Table III). No definitive correlation was observed between CXCL12 expression and progression of pancreatic cancer (Table III).

### Correlations between MVD/MLVD and clinicopathological factors of pancreatic cancer

**Figure 2:**
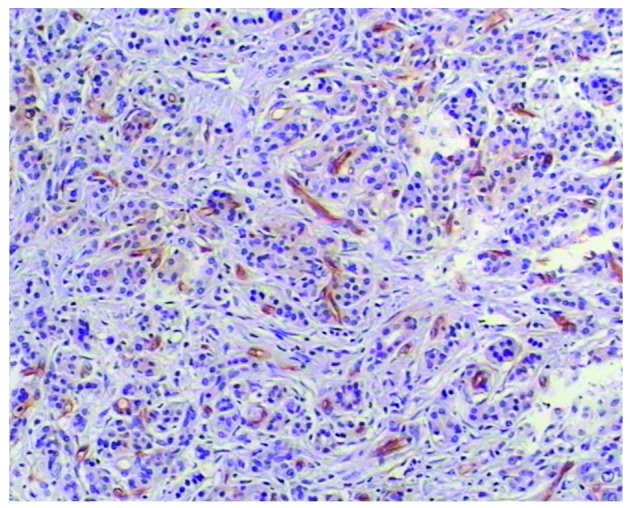
CD34-positive microvascular endothelial cells in pancreatic cancer. Magnification, ×100.

**Figure 3:**
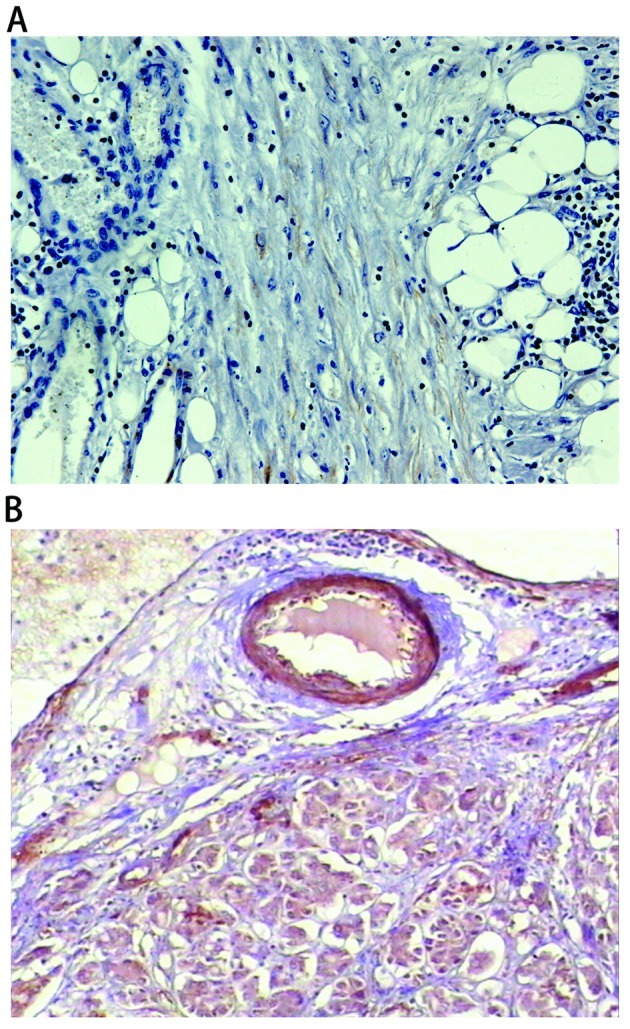
Immunohistochemical staining of VEGF-R3 to identify micro-lymphic vessel density in endothelial cells in (A) paracancerous tissues and (B) pancreatic cancer. Magnification, ×100.

Table IV shows the varying degrees of angiogenesis and lymphangiogenesis, and the relationships between these variables and clinical outcome were determined. Compared to patients with lower intratumoral MVD, patients with higher intratumoral MVD exhibited a significantly lower degree of differentiation yet a higher class of TNM staging (P<0.05). However, no relationship was found between lymph node metastasis and blood vessel formation (P>0.05). Additionally, it was found that MLVD status was correlated with tumor stage and grade. The formation of lymphatic vessels significantly increased in patients with higher TNM staging and lymph node metastasis, but no significant change was observed in patients with a lower degree of differentiation (Figs. 2 and 3).

### Analysis of the association of CXCL12/CXCR4 with MVD/MLVD

As shown in Table V, CXCL12 protein was negative in 26 samples, and the MVD in these negative samples was higher than that in the positive samples (P= 0.022). No significant correlation between the expression of CXCL12 protein and MLVD of pancreatic cancer was found (P>0.05). CXCR4 protein was positive in 24 samples, and the MLVD in these positive samples was higher than that in the negative group (P=0.003). These results indicate that lower expression of CXCL12 protein was strongly associated with angiogenesis of pancreatic cancer, while higher expression of CXCR4 protein was significantly associated with lymphangiogenesis of pancreatic cancer.

## Discussion

Chemokine molecules constitute a superfamily of inducible secreted proinflammatory proteins (11–14) that are involved in a variety of immune responses. These molecules primarily act as chemoattractants and activators of specific types of leukocytes (11,15,16), and they mediate these functions by binding to G-protein-coupled receptors. It is becoming increasingly evident that chemokines play an integral role in initiating specific immune responses (2). One such chemokine (CXCL12) is found on high endothelial venules (HEV) and within T-cell zones of the spleen and lymph nodes (3,17–19); it is involved in the recruitment of naive T cells and dendritic cells (DCs). In lymph nodes, CXCL12 plays an important role in the initiation of immune responses by co-localizing naive T cells with DC-presented antigens (20–23). The receptor for this ligand, CXCR4, is expressed on all naive T cells, some memory T cells, B cells and mature dendritic cells; it plays a central role in lymphocyte trafficking and homing to lymph nodes (24,25). Recent studies have shown the involvement of the CXCL12/CXCR4 axis in the progression of several types of cancer (4,26,27). For example, Hassan *et al* (5) reported high levels of CXCL12/CXCR4 expression in breast cancer cells and linked receptor expression to the metastatic destination of tumor cells. However, correlations between the CXCL12/CXCR4 axis and clinical features of pancreatic cancer have not been extensively studied. Therefore, in our study, we evaluated the expression of CXCL12/CXCR4 and found a critical relationship between CXCL12/CXCR4 and tumor stage and grade in pancreatic cancer.

For patients with pancreatic carcinoma, CXCL12 expression was reduced in tumor tissues, but significant levels were detected in paracancerous tissues, normal pancreas and lymph nodes. CXCR4 expression showed the opposite trend. CXCR4 was expressed in 80.0% of pancreatic carcinoma samples but only 26.7% of normal samples. In addition, we found significant differences between positive and negative CXCL12/CXCR4 samples in regards to the following clinicopathological features: i) lymph node metastasis and ii) tumor TNM staging.

To elucidate the underlying association between CXCL12/CXCR4 expression and clinicopathological features, we assessed MVD and MLVD in tumor tissues. We found that CXCL12 expression was significantly associated with the formation of blood vessels, whereas it had no obvious relationship with MLVD. The expression of CXCR4 was higher in patients with higher MLVD compared to those with lower MLVD. CXCR4 had no relationship with MVD.

Angiogenesis and lymphangiogenesis are required for many pathological processes, including tumor growth, metastasis and physiological organ/tissue maintenance. In general, the molecular mechanisms that control carcinoma progression and metastasis are related to mutations of various oncogenes, tumor-suppressor genes, metastasis-suppressor genes, and growth factors and their receptors, including Src, Ras, p16, KiSS-1, Nm23, FasL, vascular endothelial growth factor (VEGF), basic fibroblast growth factor (bFGF) and interleukin (IL)-6 (6,7,26,28). These abnormalities affect the downstream signal transduction pathways involved in the control of cell growth and other malignant properties, such as tumor staging and degree of tumor differentiation. Notably, one of the most recently recognized events in this process involves the interaction between chemokines and their receptors. Several studies have found that the chemokine receptor was highly expressed in breast and ovarian carcinomas, and the interaction between the receptor and its ligand resulted in chemotaxis, or directed migration of tumor cells from their primary site via the circulation to preferential sites of metastasis (8,29–31). These studies strongly support our hypothesis. The interaction between CXCR4 and CXCL12 may play crucial roles in the metastasis and progression of pancreatic cancer by its effects on the formation of new blood vessels and lymphatic vessels.

In conclusion, our data suggest that the chemotactic interaction between CXCR4 and its ligand CXCL12 may be a critical event in the progression of pancreatic cancer. A potential mechanism of action may be the induction of angiogenesis and lymphangiogenesis by cancer cells. This hypothesis is supported by findings that the expression pattern of CXCL12 and CXCR4 in pancreatic cancer tissue significantly correlated to clinicopathological features. Our data also suggest that the CXCL12/CXCR4 axis is associated with the formation of lymphatic vessels and blood vessels induced by pancreatic cancer cells. Additional research is underway to determine the pathways responsible for tumor cell chemokine and/or chemokine receptor-associated angiogenesis and lymphangiogenesis. It is likely that controlling such a poor prognostic feature would enable more successful loco-regional tumor control and improve survival in patients with pancreatic cancer.

## Figures and Tables

**Table I:** CXCL12 and CXCR4 expression by immunohistochemical staining in 4 groups.

	CXCL12 expression			CXCR4 expression		
Group	Negative	Positive n (%)	P-value	Negative	Positive n (%)	P-value
Cancerous tissues	26	4 (13.3)		6	24 (80.0)	
Paracancerous tissues	16	14 (46.7)	0.011	9	21 (70.0)	0.551
Normal pancreas	13	17 (56.7)	0.001	22	8 (26.7)	<0.001
Lymph nodes	15	15 (50.0)	0.006	8	22 (73.3)	0.760

**Table II:** CXCL12 and CXCR4 mRNA expression by RT-PCR.

	CXCL12 expression		CXCR4 expression	
Group	CXCL12/β-actin	P-value	CXCR4/β-actin	P-value
Cancerous tissues	0.263±0.254		0.789±0.300	
Paracancerous tissues	0.429±0.136	0.003	0.701±0.291	0.254
Normal pancreas	0.437±0.098	<0.001	0.236±0.199	<0.001
Lymph nodes	0.425±0.187	0.006	0.700±0.322	0.273
Paracancerous tissues	0.429±0.136		0.701±0.291	
Cancerous tissues	0.263±0.254	0.003	0.789±0.300	0.254
Normal pancreas	0.437±0.098	0.795	0.236±0.199	<0.001
Lymph nodes	0.425±0.187	0.925	0.700±0.322	0.99
Normal pancreas	0.437±0.098		0.236±0.199	
Cancerous tissues	0.263±0.254	<0.001	0.789±0.300	<0.001
Caracancerous tissues	0.429±0.136	0.795	0.701±0.291	<0.001
Lymph nodes	0.425±0.187	0.757	0.700±0.322	<0.001
Lymph nodes	0.425±0.187		0.700±0.322	
Cancerous tissues	0.263±0.254	0.006	0.789±0.300	0.273
Paracancerous tissues	0.429±0.136	0.925	0.701±0.291	0.99
Normal pancreas	0.437±0.098	0.757	0.236±0.199	<0.001

**Table III:** Correlation between expression of CXCL12 and CXCR4 and clinicopathological factors of pancreatic cancer.

	CXCR4 expression				CXCL12 expression		
Factors	n	Negative, n	Positive, n (%)	P-value	Negative, n	Positive, n (%)	P-value
Histology				0.311			0.810
Well-differentiated	17	5	12 (70.6)		14	3 (17.6)	
Moderately/							
Poorly differentiated	13	1	12 (92.3)		12	1 (7.8)	
TNM stage				0.050			0.324
I–II	12	5	7 (58.3)		9	3 (33.3)	
III–IV	18	1	17 (94.4)		17	1 (5.6)	
Lymph node metastasis				0.004			0.913
Negative	12	6	6 (50.0)		11	1 (8.3)	
Positive	18	0	18 (100.0)		15	3 (16.7)	

TNM, tumor node metastasis.

**Table IV:** Correlation between microvascular density/micro-lymphatic vessel density and clinicopathological factors of pancreatic cancer.

	n	Microvascular density	P-value	Micro-lymphatic vessel density	P-value
Histology			<0.001		0.194
Well-differentiated	17	54±4.3		5±5.9	
Moderately/Poorly-differentiated	13	65±6.5		8±6.4	
TNM stage			<0.001		0.017
I–II	12	52±7.4		4±2.9	
III–IV	18	63±7.3		8±4.9	
Lymph node metastasis			0.456		0.009
Negative	12	52±7.4		4±2.9	
Positive	18	54±6.9		9±5.7	

TNM, tumor node metastasis.

**Table V:** Correlation between CXCL12/CXCR4 and microvascular density/micro-lymphatic vessel density.

	n	Microvascular density	P-value	Micro-lymphatic vessel density	P-value
CXCL12			0.022		0.472
Negative	26	64±6.9		7.9±5.3	
Positive	4	55±7.0		5.9±3.1	
CXCR4			0.818		0.003
Negative	6	53±4.8		6.1±5.8	
Positive	24	55±4.1		12.1±4.0	
